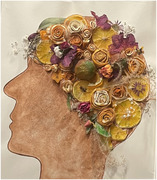# Nature and People: A Beautiful Mynd

**DOI:** 10.1002/alz70858_106140

**Published:** 2025-12-26

**Authors:** Kendra Ray, Olga Barbakova

**Affiliations:** ^1^ New York University School of Medicine, New York, NY, USA; ^2^ Menorah Center for Rehabilitation and Nursing Care, Brooklyn, NY, USA; ^3^ MJHS Menorah Center for Rehabilitation and Nursing Care, Brooklyn, NY, USA

## Abstract

**Background:**

Art has been increasingly recognized as a valuable tool in enhancing the well‐being of individuals in memory care settings. Research indicates that engaging in creative activities can significantly benefit residents with Alzheimer's disease or related dementia (ADRD) by stimulating cognitive functions, fostering social connections, and providing avenues for self‐expression. This community‐based art project was designed to explore these benefits within a skilled nursing facility, where residents could collaboratively create artwork that honors their past experiences and memories.

**Method:**

Over several weeks, a group of memory care residents (*n* = 6) at a skilled nursing facility participated in creating a piece of art titled “Nature and People: A Beautiul Mynd.” This collaborative effort involved utilizing various objects from nature including dried fruit, flowers and leaves. The process encouraged residents to tap into their long‐term memories and express themselves through the creation of nurturing and meaningful artwork.

**Result:**

A Beautiful Mynd, Memorializing Yesterdays Nurturing Dreams culminated into a visually stunning piece. The artwork is a vibrant representation of the residents' memories and experiences, as it integrates elements from nature that evoke emotional connections and reminiscence. By working together, the residents not only fostered a sense of community but also created lasting expressions of their collective memories.

**Conclusion:**

Through this project, we highlight the significant impact that community‐based art initiatives can have on enriching the lives of residents with dementia, fostering both creativity and connection.